# Dyadic Person Similarity Predicts Similarity in Face Judgements

**DOI:** 10.1177/17470218261420224

**Published:** 2026-01-23

**Authors:** Rochelle Williams, Lúcia Garrido

**Affiliations:** 1 Department of Psychology, City St George’s, University of London, UK

**Keywords:** face perception, social perception, intersubject representational similarity analysis

## Abstract

Perceivers consistently extract information from faces to judge whether others are attractive, trustworthy, or dominant. However, there is also substantial variability among perceivers when making these face-judgements. Here, we investigated whether dyadic similarities in participants’ personalities are related to similarities in their face-judgements. Three hundred and seven participants based in the United Kingdom rated 24 faces on 6 traits. Participants also rated themselves on social-traits and completed a personality questionnaire. We computed dissimilarities between pairs of participants for face-judgements, self-rated-social-traits, and personality-traits, resulting in three separate dissimilarity matrices. Using representational similarity analysis, we showed that both the self-rated-social-traits and personality-traits matrices were significantly correlated with the face-judgements matrix. Importantly, these associations were stable when controlling for age, gender, ethnicity, and location. These findings show that people who are more similar to each other also perceive others in a similar manner, and could form the basis for how we gravitate towards others and build friendships.

## Introduction

Over the past two decades, psychologists have explored the importance of face-trait perception and the potential roles it plays in our social interactions. People tend to judge very quickly whether someone has the intention and the ability to help or harm us, traits that psychologists call trustworthiness and dominance (C. A. Sutherland et al., 2013; Oosterhof & Todorov, 2008). These traits seem to be fundamental social dimensions across the world (Jones et al., 2021) as they underlie many other judgements that we make about others, particularly on first encounter. Since social interactions likely involve discerning how approachable and safe a person is, one potential characteristic of these traits is that they may be perceived in relation to the observers themselves.

Since the 1960s, researchers have investigated this relationship between the way we perceive ourselves and the way we perceive others. Dornbusch et al. (1965) found that there is more overlap in a perceiver’s use of terms to describe other people than there is when others describe a single target person. This points to the possibility that there is likely a framework that the observer is using consistently to make judgements beyond the information extracted from the face. Markus et al. (1985) found that people are more likely to attribute certain person characteristics to others if they attribute the same traits to themselves. The observed person is also perceived as more similar and more likeable by the observer. Albright et al. (1988) found significant positive correlations between people’s self-perceptions and their judgements of others based on specific characteristics such as attractiveness, formal dress sense, neatness of dressing appearance, and age (see also Srivastava et al., 2010; Wood et al., 2010). Thus, it seems likely that there is a common tendency to judge others using mental frameworks based on our own traits and dispositions. While we extract social information from the face, it is also apparent that our own dispositions play an important role in our judgements of others.

Considering this, it is important to explore the *extent* to which our own dispositions, relative to face characteristics, play a role in the way we perceive a face. Many studies have focused on the target-face characteristics that influence our social judgements. For instance, smiling faces are consistently perceived as more trustworthy and high facial masculinity is consistently perceived as more dominant (e.g., C. A. Sutherland et al., 2013; Oosterhof & Todorov, 2008; Vernon et al., 2014). However, past research has also shown that there yet remains substantial variability among perceivers when making social judgements about the same faces. Specifically, while *inter-rater* agreement (i.e., correlations between pairs of perceivers) of social judgements of faces is typically low to moderate (about .3 to .4; Hönekopp, 2006; Oosterhof & Todorov, 2008), *intra-rater* agreement (i.e., test-retest reliability of the same perceiver) is considerably higher (about .7; Hönekopp, 2006), indicating the presence of important and consistent individual differences among perceivers (see also C. A. M. Sutherland, Burton, et al., 2020; C. A. M. Sutherland, Rhodes, et al., 2020; Germine et al., 2015).

Hönekopp (2006) showed that both shared taste and private taste contributed about equally to face attractiveness judgements. More recently, Hehman et al. (2017) estimated the effects of perceiver and target variance for a large range of social traits, including intelligence, competence, attractiveness, friendliness, trustworthiness, dominance, among others. Results from multilevel models quantified the variance that was attributable to the perceiver and to the target, and the authors found that the variance in ratings for all traits was due to *both* the perceiver and the target. In other words, raters consistently relied on factors related to themselves and on factors related to the target face characteristics, and the extent of the relative contributions of the perceiver and target depended on the trait.

Given that much variance in face judgements is related to the perceiver, this variance could be related to our individual differences. Face judgements are indeed influenced by the particular groups or subgroups to which the perceivers belong, and are thus affected by ethnicity, gender (Hönekopp, 2006), and cultural background (Sofer et al., 2017). Face judgements are also influenced by individual differences in other dispositions such as anxiety levels (Willis et al., 2013) and personality traits (Mattarozzi et al., 2015). For example, Mattarozzi et al. (2015) found that those higher in trait agreeableness perceived faces as more trustworthy.

A recent study investigated the relationship between the structure of personality and face perception across the world (Oh et al., 2022). Specifically, the authors investigated whether the personality structure of people living in one region relates to how they perceive social traits in the faces of others. Self-report personality trait ratings and face trait judgements were gathered from large existing datasets from 42 countries. Oh et al. (2022) computed average distances between trait pairs, separately for personality and face judgements. These resulted in representational dissimilarity matrices (RDMs) of the structure of both personality and face judgements for each world region. Results showed that, for most world regions, the two RDMs were significantly associated, indicating that the structure of personality was related to the structure of face perception. In other words, where there is a co-occurrence of traits in one’s personality, this co-occurrence is also reflected in the social judgements made about the face. Furthermore, two countries that were more similar in their personality structure also showed higher similarity in the structure of face judgements.

It is possible that, even within a single world region, people who are more similar to each other in their own dispositions and traits also show more similar social trait judgements. Support for this comes from recent neuroimaging studies that have shown that pairs of people with similar personality traits also have similar brain activation patterns. Finn et al. (2020) investigated the relationship between pairwise brain similarity and personality similarity, using inter-subject representational similarity analysis (RSA; Kriegeskorte et al., 2008). The authors analysed functional magnetic resonance imaging (fMRI) data of participants who viewed naturalistic video-clips, and personality ratings from the same participants. Results showed that inter-subject similarities in personality between participants were associated with inter-subject similarities in brain activity in ventral temporal cortex, frontal cortex, and cerebellum. Matz et al. (2022) found comparable results using both fMRI and electroencephalography. These results suggest that an alignment in brain activity can indeed be predicted by an alignment in personality.

Successful social interactions between people partly rely on a consistent understanding of our social world, so the alignment between people with similar dispositions may lead to successful social interactions, such as the initial stages of friendship development, affiliation, motivations towards community formation, and relevant in-group behaviours (Robbins & Krueger, 2005). Selfhout et al. (2010) showed that people choose others as friends who are similar to themselves in levels of agreeableness, extraversion, and openness. Parkinson et al. (2018) have also demonstrated that similarities in brain activity can predict friendship and social distance between people. In terms of trait perception, Bronstad and Russell (2007) showed that pairs of people in close relationships have higher inter-rater agreement when rating face attractiveness compared to pairs of strangers. However, it was not possible from this study to distinguish whether these shared perceptions resulted from spending time together and having shared experiences, or whether the preferences themselves contributed to the formation of the relationships. In any case, it seems possible that the motivation to gravitate towards those who are like us and become friends may be related to an alignment in perspectives.

Taken together, the studies reviewed here suggest that there is an important link between how similar we are to others and the effect on any shared perspectives we might have. However, there remains the question as to the specific content of these aligned perspectives, especially relating to social perception. Specifically, do we perceive people in a similar way if we have more similar personalities? The present study aims to investigate whether individuals who are more similar in their personality and self-perceived social trait dispositions also perceive face traits in a more similar manner. We used inter-subject RSA (Finn et al., 2020; Kriegeskorte et al., 2008) to analyse the association between person similarity and face judgement similarity. We hypothesised that pairs of people who are more similar in their own (self-perceived) personality and social traits also make judgements about faces in a more similar way. More specifically, we predicted that intersubject person similarity (measured as similarity in personality-traits and similarity in self-rated-social-traits) is significantly positively correlated with face-judgement similarity (based on ratings of faces).

## Methods

### Participants

We recruited 320 participants in total (*M*_age_ = 37.88; *SD* = 11.54; 182 identified as women, 133 as men, and 4 as other). We pre-registered our study, specifying sample size, inclusion, and exclusion criteria (https://doi.org/10.17605/OSF.IO/ZR92G). Participants were recruited through the online participant pool Testable Minds (https://minds.testable.org) and received compensation for their time. Our goal was to test 300 participants. We stipulated that if, after applying the exclusion criteria, the sample size fell below 300 participants, we would test additional participants in batches of 10 until a final sample of at least 300 was obtained.

As inclusion criteria, all participants had to be aged 18 years or older, have English as their first language, and be living in the United Kingdom. Given that Oh et al. (2022) found an effect of country on the perception of social traits, here we wanted to avoid country as a source of variability and therefore focused on people in the United Kingdom only.

We had three pre-registered exclusion criteria to ensure data quality. First, there were four ‘catch’ trials, and participants who failed two or more catch trials were excluded. The catch trials are described in the ‘Procedure’ section. Second, we excluded participants who provided the same rating in 10 consecutive trials of the Face Judgements Task. Third, we excluded participants who responded too quickly (responses of 200 ms or less) in 10% or more of the trials of the Face Judgements Task. The exclusion criteria were applied before analysis, and 13 participants were excluded. Two participants were excluded because of the first and second criteria, and 11 participants were excluded because of the second criterion only. No participants were excluded because of the third criterion.

The final sample consisted of 307 individuals (*M*_age_ = 37.97; *SD* = 11.55; 171 women, 132 men, 4 other). Two hundred and sixty-eight participants were right-handed, 29 were left-handed, and 10 were ambidextrous. Two hundred and thirty-eight participants described their ethnicity as White, 12 as Mixed, 27 as Asian, 25 as Black, and 5 as ‘Other’ (we used these ethnicity terms based on options from the 2021 census; Office for National Statistics, 2021). All participants lived in the United Kingdom and reported their geographical location (see Supplemental Material 1).

The study was approved by the Psychology Research Ethics Committee at City, University of London.

### Design and Variables

We used a correlation design with two main variables: person similarity and face-judgement similarity. We operationalised person similarity in two ways (a) Personality-traits similarity: the similarity between participants in terms of their Big Five personality traits, which were measured with the Personality-traits Questionnaire described below, and (b) Self-rated-social-traits similarity: the similarity between participants in terms of self-perceived social traits, which were measured with the Self-rated-social-traits Questionnaire described below. Face-judgements similarity corresponds to the similarity between participants in terms of their judgements of social traits in faces of others, measured with the Face-Judgements Task described below.

### Materials

#### Personality-Traits Questionnaire

We used the International Personality Item Pool (IPIP-NEO-60) personality questionnaire to measure personality-traits (Maples-Keller et al., 2019). The IPIP-NEO-60 has shown to have strong internal consistency and validity when compared against questionnaires such as the NEO PI–R (Costa & McCrae, 1992; Maples-Keller et al., 2019). It consists of 60 statements, such as ‘Love to daydream’. Participants rated how well each statement described them using a 5-point scale ranging from 1 (*Very inaccurate*) to 5 (*Very accurate*). There are 12 items that correspond to each of the 5 Big Five personality traits (Openness, Conscientiousness, Extraversion, Agreeableness, and Neuroticism).

#### Self-Rated-Social-Traits Questionnaire

We created a questionnaire to measure self-rated social traits. We used 13 traits used by Oosterhof and Todorov (2008): Trustworthiness, Dominance, Attractiveness, Sociability, Emotional Stability, Aggressiveness, Intelligence, Care, Meanness, Confidence, Weirdness, Unhappiness, and Responsibility. Participants were asked to ‘Please rate how well each word describes YOU’. Each word was presented next to a 9-point scale from 1 (*Does not describe me*) to 9 (*Describes me*).

#### Face-Judgements Task

Stimuli consisted of 24 neutral face stimuli from the Chicago Face Database (https://www.chicagofaces.org; Ma et al., 2015, 2021). Six faces per ethnicity were selected (three men and three women). Faces from four ethnicities were selected: White, Black, Latino, and East Asian. Supplemental Material 2 has a list of the names of the pictures selected from the database. The pictures had white backgrounds and were presented in colour with a 600 × 450-pixel size.

### Procedure

The study was created on Testable (https://www.testable.org; Rezlescu et al., 2020). Participants completed the study online on a desktop or laptop computer. Participants read the information sheet and provided consent to take part in the study. Participants then completed the Face-judgements Task, followed by the Self-rated-social-traits Questionnaire and finally the Personality-traits Questionnaire.

In the Face-judgements Task, participants were presented with the face pictures and were asked to judge each face on six social traits. The traits were: Trustworthiness, Dominance, Attractiveness, Sociability, Aggressiveness, and Intelligence. These traits are known to be associated with the main dimensions in which we judge the faces of others (Sutherland et al., 2013; Oosterhof & Todorov, 2008) and were shown to explain a substantial part of the variance in social judgements ratings of participants in the United Kingdom (Jones et al., 2021). In each trial, participants were presented with one face paired with one trait and were asked to rate the face, for example, ‘How TRUSTWORTHY is this person?’ on a 9-point scale from 1 (*Not at all*) to 9 (*Extremely*). Each of the 24 faces was paired with each of the traits, resulting in 144 total trials. The order of trials was randomised for each participant.

A total of four attention quality check trials or ‘catch’ trials were included in the study and were related to the Face-judgements Task. For the first attention check, after reading the instructions of the face-judgements task but before starting the task, participants were presented with a statement which read ‘Please confirm what you will be doing in the present study’. Participants selected out of three simple options: ‘rating places’, ‘rating faces’, and ‘rating objects’. The remaining attention check trials were presented at the end of the Face-judgements Task: these tested whether participants remembered which faces they had seen during the task. We reasoned that if participants had been paying attention, they would be able to distinguish faces that were presented during the task (each repeated six times) from novel faces. There were three trials. In each trial, three faces were presented, where two of the faces had been shown in the face-judgements task and one face was novel. Participants had to select the novel face. The three faces within each trial were of the same ethnicity and gender (e.g., three white males, three black women, three Latino males).

Participants then completed the Self-rated-social-traits Questionnaire and finally they completed the Personality-traits Questionnaire. Upon finishing the study, participants were prompted to read the study debrief and click ‘FINISH’ to save their results.

### Data Analysis

We used inter-subject RSA (Finn et al., 2020; Kriegeskorte et al., 2008) to analyse the association between person similarity and face-judgements similarity. RDMs were computed, where each entry in a matrix represents the pairwise dissimilarity between two participants on each of our measures. Three main RDMs (each with dimensions 307 × 307) were computed: (1) RDM Personality-traits: each participant’s scores on the Personality-traits Questionnaire were compared with each of the other participants’ scores in a pairwise way (i.e., each matrix entry contains the dissimilarity value between pairs of vectors, with each vector containing 60 items), (2) RDM Self-rated-social-traits: each participant’s scores on the Self-rated-social-traits Questionnaire were compared with each of the other participants’ scores in a pairwise way (i.e., each matrix entry contains the dissimilarity value between pairs of vectors, with each vector containing 13 items), and (3) RDM Face-judgements: each participant’s ratings on the Face-judgements Task were compared with each of the other participant’s scores in a pairwise way (i.e., each matrix entry contains the dissimilarity value between pairs of vectors, with each vector containing 144 items). We used Euclidean distance as the main measure of dissimilarity in the RDMs. We first *z*-scored the vectors for each participant before computing Euclidean distances. This is important so that the dissimilarities between individuals consist of the distances between patterns of ratings and not distances in overall mean ratings between participants.

For the main analyses, we first correlated RDM Self-rated-social-traits with RDM Face-judgements and second, we correlated RDM Personality-traits with RDM Face-judgements. Before computing correlations, we vectorised each of the 307-by-307 RDMs by extracting the lower off-diagonal (i.e., all elements below the main diagonal) of each matrix. This yielded 3 vectors (one for each RDM) containing 46,971 values each. We used Spearman rank order correlations of the resulting vectors. For readability, we describe below that we correlated RDMs, but in all cases, we correlated the vectorised RDMs. Because the entries in each matrix (and values of resulting vectors) are not independent from each other (i.e., the same participant appears paired with each of the other participants), we cannot use parametric statistics, which rely on independence assumptions (Chen et al., 2016; Selfhout et al., 2009). We thus tested whether each of these two correlations was significantly higher than zero by using random permutation tests. To do this, we conducted 1,000 iterations. For each iteration, we re-computed the Person Similarity RDM by first randomly permuting the order of participants and then re-computing the RDM. Then, we computed the correlation between the permuted person similarity RDM (RDM Self-rated-social-traits or RDM Personality-traits) and the original RDM Face-judgements to obtain a correlation for that iteration. This allowed us to estimate the null distribution with 1,000 permuted correlations. To be considered significant, the ‘actual’ correlation needed to be higher than 95% of the ‘permuted’ correlations (*p* < .05).

We also computed confidence intervals of the correlations by bootstrapping (random resampling with replacement). For each of 1,000 resamples, we randomly selected 307 participants (resampling with replacement), re-computed all RDMs, and conducted all correlation analyses again. We provide the 95% confidence intervals of the correlations based on the 2.5th and 97.5th percentiles of the bootstrapped correlations.

All analyses (except mixed effects modelling) were conducted in Matlab (version R2021b, www.mathworks.com; Mathworks, Natick, Massachusetts). All anonymised data and code for main analyses are openly available: https://doi.org/10.25383/city.26519890.

## Results

**Figure 1.**
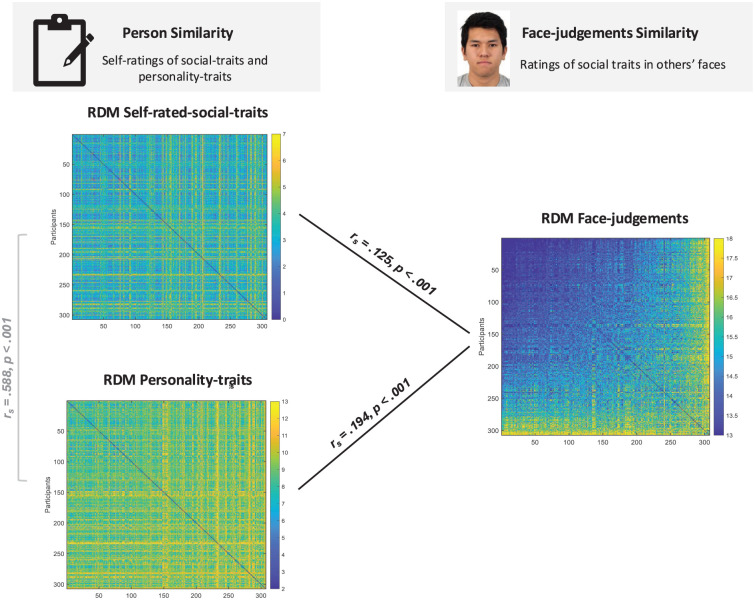
Intersubject RDMs depicting the pairwise (dis)similarity between all participants (Self-rated-social-traits similarity, Personality-traits similarity, Face-judgements similarity) and their correlations. *Note.* Intersubject RDMs for each variable and Spearman correlations between RDMs: RDM Self-rated-social-traits, RDM Personality-traits, RDM Face-judgements. Colour bars show Euclidean distances with blue indicating higher similarity (lower distance) and yellow indicating lower similarity (higher distance). RDM Face-judgements is ranked for visualisation purposes only, depicting the most similar pairs of participants from the top left of the matrix (i.e., darker blue entries) to the least similar pairs of participants at the bottom right of the matrix (i.e., yellower entries). The other RDMs are ranked based on this same RDM. Spearman correlations are shown between the vectorised RDMs, with *p*-values estimated with random permutation testing. RDM = representational dissimilarity matrix.

In this study, we aimed to investigate whether person similarity is related to face-judgements similarity. In other words, do people who are more similar to each other in terms of their dispositions also make more similar face judgements? In the Face-judgements Task, participants rated 24 faces, each face on 6 social traits (trustworthiness, dominance, attractiveness, sociability, aggressiveness, and intelligence). To measure their own dispositions and personality traits, participants completed a Self-rated-social-traits Questionnaire, and a Personality-traits Questionnaire. For each of the three variables, we computed pairwise similarity for all pairs of participants. Briefly, we computed vectors of all *z*-scored ratings for each participant. We then computed Euclidean distances between vectors of all pairs of participants (for the same variable) to obtain pairwise distances (dissimilarities) between participants. This resulted in one intersubject RDM for each of the three variables: RDM Face-judgements, RDM Self-rated-social-traits, and RDM Personality-traits (Figure 1). We then investigated whether intersubject person similarity could predict intersubject face-judgements similarity.

### Person Similarity Is Associated with Face-Judgements Similarity

#### Correlation Between Self-Rated-Social-Traits Similarity and Face-Judgements Similarity

The first analysis looked at whether similar people (in terms of their self-rated social-traits) also make similar judgements of social traits in faces. To do this, RDM Self-rated-social-traits was correlated with RDM Face-judgements using Spearman rank-order correlation (after vectorising the elements below the main diagonal of each matrix), resulting in a positive correlation of *r_s_* = .125 (95% bootstrapped confidence interval [0.069, 0.198]; Figure 1). We carried out non-parametric permutation tests to estimate a null distribution and test whether this correlation was significantly higher than zero. For each of 1,000 iterations, the RDM self-rated-social-traits was re-computed after randomly shuffling (permuting) the order of participants, and then we re-computed the correlation between the original RDM Face-judgements and the permuted RDM Self-rated-social-traits. To be considered significant, the actual correlation had to be higher than 95% of the permuted correlations. Results showed that the actual correlation was higher than all 1,000 permuted correlations, and therefore was significantly higher than zero (*p* < .001, permutation tests). In other words, the more similar pairs of people were in the ratings of their own social traits, the more similar their ratings were of the faces of other people, which supports our hypothesis.

#### Correlation Between Personality-Traits Similarity and Face-Judgements Similarity

The same procedure was carried out: RDM Personality-traits was correlated with RDM Face-judgements (after vectorising the RDMs). There was a positive correlation of *r_s_* = .194 (95% bootstrapped confidence interval [0.139, 0.262]), which was significantly higher than zero (*p* < .001; Figure 1). These results revealed that the more similar people are in their personality structure, the more likely they are to rate faces in a similar way, which supports our hypothesis. For completeness, we also correlated the RDM Self-rated-social-traits with the RDM Personality-traits (see Supplemental Material 3).

#### Split-Half Reliability of Face-Judgements Similarity

We also computed split-half reliability of the RDM Face-judgements as a way of estimating the highest possible correlation we could expect with face-judgements similarity (i.e., as a way of estimating the noise ceiling). To compute split-half reliability, we conducted 1,000 random splits of the Face-judgements Task data and re-computed the RDM Face-judgements (again, 307 × 307 RDM across participants) for each split, and then correlated the RDMs across splits. More specifically, for each iteration, we randomly divided the data on the Face-judgements Task for each participant in 2 halves (with 72 trials in each half). We then computed an RDM Face-judgements using all data from split 1 (a 307 × 307 RDM across participants) and an RDM face-judgements using all data from split 2 (again, a 307 × 307 RDM across participants). In other words, the RDMs for each split have the same structure as the RDM Face-judgements for the main analysis, but they are based on just half of the rating data from each participant. For each iteration, we correlated the two RDMs (split 1 vs. split 2; after vectorising the values under the main diagonal for each RDM) using Spearman correlations. The resulting correlations across 1,000 iterations varied between .474 and .605, with a mean of *r_s_* = .558. This is our estimation of the reliability of RDM Face-judgements and shows moderate consistency between participants’ differences when using independent data across splits. We also interpret this reliability as the maximum correlation we could expect of any variable or model with the RDM Face-judgements. When considering this noise ceiling, the person similarity variables seem to explain a substantial proportion of the variance in face-judgements similarity (22.40% for RDM Self-rated-social-traits and 34.77% for RDM Personality-traits), though there is still considerable unexplained variance in face-judgements similarity.

### Person Similarity Is Associated with Face-Judgements Similarity After Controlling for Demographic Variables

We next investigated whether the positive correlations between person similarity and face-judgements similarity could be accounted by similarities in demographic variables. In other words, we investigated whether the correlations between person similarity and face-judgements similarity could be accounted by similarities in the demographic variables age, gender, ethnicity, and participant geographical location. We thus carried out multiple regression analyses with RDM Face-judgements as the outcome variable and person similarity as predictor variables, controlling for age, gender, ethnicity, and participant location. We note that we use predictor and outcome variables here solely as statistical terms for the multiple regression, in that the variance of one variable can predict the variance of another variable. Our study design is correlational and from our data, we cannot establish causality or determine which variables precede others. As before, and because the entries in each RDM are not independent, we conducted permutation tests to test whether parameter estimates from the regression analyses were significantly different from zero. *p*-values for the beta-values were estimated using random permutations (1,000 iterations) to estimate null distributions of the parameter estimate for each variable.

RDMs were computed for each demographic variable (RDM Age, RDM Gender, RDM Ethnicity, and RDM Region). Pairwise distances between each pair of participants were computed based on each demographic variable separately, thus producing a dissimilarity matrix for each variable (i.e., for RDM Age, pairwise distances in age were computed between all participants). RDM Age used Euclidean distance as the dissimilarity measure. For RDM Gender and RDM Ethnicity (which were based on categorical variables), we classified each pair of participants as same (‘0’) or different (‘1’). For RDM Region, the actual geographical distances between latitude and longitude co-ordinates for the centre of each U.K. region were computed using Haversine distance. Co-ordinates for the midpoint of the 12 U.K. regions were obtained using GeoMapApp (www.geomapapp.org; Ryan et al., 2009).

#### Multiple Regression Predicting Face-Judgements Similarity with Self-Rated-Social-Traits Similarity and Demographic Variables as Predictors

We first looked at the association between RDM Self-rated-social-traits and RDM Face-judgements (after vectorising the RDMs by extracting the values below the diagonal). As expected, simple regression analysis showed that self-rated-social-traits similarity could significantly predict face-judgements similarity (β = .123, *p* < .001, permutation tests), explaining 1.78% of the variance (*R*^2^ = .0178). When all RDMs for demographic variables were included as predictors along with the RDM Self-rated-social-traits (all RDMs vectorised), the total model explained 6.13% of the variance in face judgement similarity (*R*^2^ = .0613). Looking at the parameter estimates for each predictor variable (Table 1), similarity in all demographic variables was associated with face-judgements similarity (especially ethnicity), but there is unique substantial variance explained by person similarity (β = .138, *p* < .001).

**Table 1.** Regression Analyses Using Self-Rated-Social-Traits Similarity to Predict Face-Judgements Similarity.

Predictor variables	Simple regression, β, *p*	Multiple regression, β, *p*
Self-rated-social-traits similarity	.123, <.001	.138, <.001
Age		.065, .007
Gender		.033, .013
Ethnicity		.184, .001
Region		.082, .036

*Note.* Simple regression results including only RDM Self-rated-social-traits as predictor. Multiple regression results including RDM Self-rated-social-traits and RDMs for demographic variables as predictors: β = parameter estimates; *p*-values estimated by random permutations. RDM = representational dissimilarity matrix.

#### Multiple Regression Predicting Face-Judgements Similarity with Personality-Traits Similarity and Demographic Variables as Predictors

We next looked at the association between RDM Personality-traits and RDM Face-judgements (after vectorising the RDMs by extracting the values below the diagonal). Simple regression showed that personality-traits similarity could predict face-judgements similarity (β = .199, *p* < .001, *R*^2^ = .0397). When all the RDMs for demographic variables were included as predictors along with the RDM Personality-traits, the model explained 7.8% of the variance in face-judgements similarity (*R*^2^ = .078). Looking at the parameter estimates for each predictor variable (Table 2), all RDMs for demographic variables were associated with face-judgements similarity (especially ethnicity), but there is unique substantial variance explained by personality-traits similarity (β = .190, *p* < .001).

**Table 2.** Regression Analyses Using Personality-Traits Similarity to Predict Face-Judgements Similarity.

Predictor variables	Simple regression, β, *p*	Multiple regression, β, *p*
Personality-traits similarity	.199, <.001	.190, <.001
Age		.059, .017
Gender		.031, .014
Ethnicity		.170, <.001
Region		.085, .032

*Note.* Simple regression results including only RDM Personality-traits as predictor. Multiple regression results including RDM Personality-traits and RDMs for demographic variables as predictors. β = parameter estimates; *p*-values estimated by random permutations. RDM = representational dissimilarity matrix.

#### Linear Mixed Effects Modelling with Crossed Random Effects

To address the non-independence issues of intersubject correlation analyses when controlling for covariates, Chen et al. (2017) recommended the use of linear mixed effects modelling with crossed random effects to account for repetitions of participants in multiple dyads. Therefore, in addition to the multiple regression analyses described above, we also conducted linear mixed-effects modelling. We used R Statistical Software (version 4.4.2; R Core Team, 2024). We followed the procedures suggested by Chen et al. (2017; doubling the data, correcting for degrees of freedom) and fit linear mixed models using lme4 (version 1.1.35.5; Bates et al., 2015) and lmerTest (version 3.1.3; Kuznetsova et al., 2017). We thus used linear mixed effects models to predict face-judgements similarity with person similarity (separate models for self-rated-social-traits similarity and personality-traits similarity) and demographic variables as fixed effects, and participants in each dyad as random effects. Results showed that self-rated-social-traits similarity was a significant predictor of face-judgements similarity (β = .076, *t* = 21.840, *p* < .001). Personality-traits similarity was also a significant predictor of face-judgements similarity (β = .072, *t* = 23.61, *p* < .001). These results are in line with the multiple regression results above and again show that person similarity can predict face-judgements similarity, even after controlling for demographic predictors.

### Results Are Stable with At Least 120 Participants and Across Traits

We next conducted exploratory analyses to investigate the stability of the above results. We first investigated the stability of the main correlations between person similarity and face-judgements similarity across multiple sample sizes and found that correlations between RDMs became stable with around 120 participants (see Supplemental Material 4). This analysis also demonstrated that the results are not just dependent on our large sample size, nor are they dependent on only a few participants.

We next investigated whether the association of person similarity with face-judgements similarity is stronger for some face judgements than others. Results showed that person similarity was significantly associated with face-judgements similarity for all traits separately, though all correlations were lower than when considering all traits together (see Supplemental Material 5).

We also conducted exploratory analyses investigating the extent to which individual differences in specific social and personality traits contribute to *average* face judgements (see Supplemental Material 6). These results suggested that our main findings of similarity in face-judgements similarity being predicted by person similarity cannot be fully explained by associations of any specific personality traits (or self-rated-social-traits) with means for specific face-judgements and instead seem to reflect *patterns* of responses across traits. Finally, we computed inter-rater agreement of face judgements to investigate agreement of face-judgements across participants (see Supplemental Material 7). Results showed low to moderate inter-rater agreement, which is comparable to results from previous studies (e.g., Oosterhof & Todorov, 2008).

## Discussion

In the present study, we investigated whether people who are similar in their personality and social dispositions also make more similar social judgements about others. We measured pairwise similarity between participants’ traits and dispositions (i.e., person similarity) using their responses from self-report questionnaires on social traits (self-rated-social-traits) and the Big Five personality traits (personality-traits). We separately looked at the similarity between participants’ face judgements using their ratings of unfamiliar faces based on six social traits (trustworthiness, dominance, attractiveness, sociability, aggressiveness, and intelligence). We found that there was a small but reliable correlation between pairwise person similarity and pairwise face-judgements similarity. Thus, two people who perceive themselves in a similar way, in terms of their self-rated-social-traits as well as their personality-traits, also make more similar social judgements about others’ faces. This effect is present even after controlling for age, gender, ethnicity, and geographical location (though, these variables, especially ethnicity, also contribute to a significant proportion of independent variance found in face-judgements similarity). The results suggest that pairs of people who are similar in their own traits and dispositions *perceive* others in a similar manner.

The results from this study provide support for our hypothesis and are consistent with results from previous studies (Finn et al., 2020; Matz et al., 2022; Oh et al., 2022). In particular, Oh et al. (2022) demonstrated that the personality structure within a given world region is related to the structure of face perception within that same region. In our study, we extended these findings to show that these associations are not dependent on world region only and can be seen within the same country. Thus, within the United Kingdom only, pairs of people who have similar personalities also perceive others’ faces in a more similar way. Therefore, individuals’ own personalities and dispositions (within the same culture and speaking the same native language) are also related to how they perceive faces. While this study was designed to look within one country in particular, it is an obvious limitation that the results cannot be generalised to different countries/regions/cultures. It is important for future studies to replicate this effect within different countries, world regions, and cultures.

Finn et al. (2020) and Matz et al. (2022) demonstrated that those with similar personalities also had more similar brain activity. Here, we extended these findings to show that similarity in personality and social traits is also related to their social perception, specifically in participants’ first impressions of faces. It would be interesting to investigate in future studies whether similarity in dispositions predicts similarity in other behaviours as well, and specifically if the association observed here depends more strictly on using social stimuli such as faces.

Despite much focus of social trait perception research on the characteristics of the target faces that are associated with different types of judgements, there is increasing evidence showing that the *perceiver* characteristics also contribute to the variance in social trait judgements of faces (e.g., Hehman et al., 2017; Hönekopp, 2006). We believe that our study contributes to the understanding of the sources of this variance due to the perceiver. We suggest that there are systematic sources of variance in face trait ratings related to participants’ own traits and dispositions, and that these can be studied by looking at covariance/similarities across participants.

Many studies in the field of person perception have reported substantial perceiver effects when rating others (e.g., Albright et al., 1988; Srivastava et al., 2010; Wood et al., 2010). These studies have focused on ratings of others’ personality-traits (many times after meeting others in-person) and not on ratings of social traits in faces per se but have consistently shown that individuals tend to perceive others in a particular way that relates to their own personality. In some cases, individuals tend to perceive others with similar traits as they perceive themselves, but this is not always the case (Wood et al., 2010). These past studies, however, have focused on global effects of perceiving others: the same individual tends to perceive others as generally more (or less) agreeable, for example. Our study suggests an additional dimension to these perceiver effects, in that the individual’s own personality-traits are associated with *which* specific faces are perceived to be more or less trustworthy, for example. It is not a global effect of finding all faces more (or less) trustworthy, but instead how perceivers rank specific faces on trustworthiness (i.e., what is the specific pattern of ratings of the faces). In other words, the perceiver’s personality traits seem to be associated with particular ways in which they map facial characteristics to social-traits ratings. It will be very interesting for future studies to attempt to bring these fields together to fully understand how perceiver effects contribute to social and person perception.

We believe that there are several possible mechanisms that could explain the link between person similarity and face-judgements similarity. One possible mechanism could be that individuals with similar personality traits may look at faces in a similar way and attend to similar features. Peterson and Eckstein (2013) reported substantial individual differences in the preferred point of fixation when encountering a face: perceivers showed idiosyncratic eye movement patterns that were consistent across stimuli and time. Presumably, if people tend to focus on different parts of the face, they could make different judgements about faces. Future studies could investigate whether individuals with similar personality traits may have similar eye movement patterns when looking at faces and also investigate whether individuals with similar eye movement patterns may judge faces in a similar way. A second possible mechanism explaining the association of person similarity with face-judgements similarity could be that participants with similar personality traits may share similar implicit or lay theories about how face characteristics and judgements about traits relate to each other. In other words, they may have similar ways of mapping face space (face features, visual properties of faces) to trait space (judgements about traits; Over & Cook, 2018). This mapping of face space to trait space could be learned over time, but it would be interesting to investigate if individuals with similar personality traits have similar mappings between face space and trait space.

In terms of the implications of our results, we propose that the similarities between participants’ traits and dispositions could be relevant to how people form friendships and relationships. One on hand, Selfhout et al. (2010) showed that friends choose others who are similar to themselves in levels of agreeableness, extraversion, and openness. On the other hand, Bronstad and Russell (2007) showed that pairs of friends (or people in close relationships) rate attractiveness in faces in a similar manner. These similarities in social perception could happen because friends spend time together and develop shared views. However, our results showed that even strangers who are similar in terms of their personality and social traits also perceive others in a similar way. We suggest that this could be an important mechanism in the formation of friendships: maybe we gravitate towards similar others because we actually perceive the social worlds as they do. If people with similar dispositions also perceive others in a similar way, it seems possible that they will trust and approach the same people. It would be interesting to test this directly in small-scale communities while they are forming (Parkinson et al., 2018).

One important limitation of our results is that we only know that person similarity is *associated* with face-judgements similarity but cannot know about causality or how this association emerged/developed. It is also possible that a third variable is associated with both person similarity and face-judgements similarity, but that there is no mechanistic link between the two variables per se. We think future studies replicating this association across regions/samples, and also looking at possible third variables, will be very valuable. Similarities in upbringing, environments, culture (Sofer et al., 2017), mood, and even cognitive domains could contribute to the association between person similarity and face-judgements similarity. We think that larger studies looking at a broader range of variables, and even across development, could help shed light on the possible mechanisms linking these variables.

The effect size of the association between person similarity and face-judgements similarity was small, with correlations of .13 and .19. We argue, however, that these effect sizes were stable across different analyses, and when using sub-samples of the data (see Supplemental Material 4). We also note that our estimated noise ceiling for the RDM Face-judgements was around .56, which we interpret as the maximum correlation we could expect with this variable. In this light, the observed correlations show that person similarity can explain about 22% to 35% of the RDM Face-judgements. Even though these are not negligible effects, there is still considerable unexplained variance in face-judgements similarity.

One final important limitation is that we did not control for the possibility of participants knowing each other. Given that our data was collected online, it was impossible for us to obtain such information. In a country of more than 60 million people, it seems unlikely that many participants knew each other. Future studies could record IP addresses or latitude/longitude for more accurate determination of location and even recruit an equal number of participants from each region. It could also be interesting for future studies to directly manipulate whether participants know each other or not.

To conclude, we investigated the relationship between dyadic person similarity and the similarity in face judgements. We showed that pairs of people who are similar in their dispositions and personalities also make more similar judgements about others’ faces. Additionally, those who were of the same gender and ethnicity, and those who were nearer in terms of age and geographical location also made more similar judgements, but these effects were independent from the one accounted for by person similarity. Future studies could investigate whether the association between person similarity and face-judgements similarity is important for how people build friendships.

## Supplemental Material

sj-docx-1-qjp-10.1177_17470218261420224 – Supplemental material for Dyadic Person Similarity Predicts Similarity in Face Judgements Supplemental material, sj-docx-1-qjp-10.1177_17470218261420224 for Dyadic Person Similarity Predicts Similarity in Face Judgements by Rochelle Williams and Lúcia Garrido in Quarterly Journal of Experimental Psychology
