# Supplementary material for: Dyadic Person Similarity Predicts Similarity in Face Judgements
Source: Q J Exp Psychol (Hove). 2026 Jan 23;79(8):2199–210. doi: 10.1177/17470218261420224 (PMC13400793; doi:10.1177/17470218261420224)
Supplement: sj-docx-1-qjp-10.1177_17470218261420224 – Supplemental material for Dyadic Person Similarity Predicts Similarity in Face Judgements [file sj-docx-1-qjp-10.1177_17470218261420224.docx]

**SUPPLEMENTARY INFORMATION**

**Dyadic Person Similarity Predicts Similarity in Face Judgements**

Rochelle Williams^1^, Lúcia Garrido^1^

^1^ Department of Psychology, City St George’s, University of London

**Supplementary Information 1**

**Participants’ regions of residence in the United Kingdom**

All participants lived in the UK and reported their geographical location (see Table S1; we used UK regions based on Census 2021 data published from the Office of National Statistics: <https://explore-local-statistics.beta.ons.gov.uk/areas/K02000001>).

**Table S1**

*Participants’ regions of residence in the United Kingdom*

|  |  | Region | N Participants |
| --- | --- | --- | --- |
|  |  | North East | 7 |
|  |  | North West | 35 |
|  |  | Yorkshire and The Humber | 23 |
|  |  | East Midlands | 17 |
|  |  | West Midlands | 29 |
|  |  | East of England | 31 |
|  |  | London | 56 |
|  |  | South East | 37 |
|  |  | South West | 22 |
|  |  | Wales | 13 |
|  |  | Scotland | 32 |
|  | **Total** | Northern Ireland  12 | 5  307 |

*Note.* Number of participants living in each of twelve UK regions (taken from the Census 2021; the Office of National Statistics).

**Supplementary Information 2**

**List of pictures used from the Chicago Face Database used in the Face-judgements Task**

CFD-AF-203-077-N

CFD-AF-204-067-N

CFD-AF-206-079-N

CFD-AM-225-102-N

CFD-AM-201-076-N

CFD-AM-203-086-N

CFD-BF-027-002-N

CFD-BF-002-001-N

CFD-BF-007-001-N

CFD-BM-009-002-N

CFD-BM-027-001-N

CFD-BM-227-191-N

CFD-LF-204-133-N

CFD-LF-208-127-N

CFD-LF-215-157-N

CFD-LM-210-156-N

CFD-LM-214-165-N

CFD-LM-219-295-N

CFD-WF-001-003-N

CFD-WF-008-002-N

CFD-WF-011-002-N

CFD-WM-018-002-N

CFD-WM-025-002-N

CFD-WM-200-034-N

**Supplementary Information 3**

**Correlation between Self-rated-social-traits similarity and Personality-traits similarity**

For completeness, we correlated the RDM Self-rated-social-traits with the RDM Personality-traits. This would inform us about the consistency in self-report person similarity between participants. Results showed that there was a positive and substantial correlation between the two RDMs (*r_s_* = .588; 95% bootstrapped CI = [.537 .641]), which was significantly higher than zero (*p* < .001, permutation tests). These results suggest that there is high consistency in the two self-report person similarity measures.

**Supplementary Information 4**

**How many participants are needed for stable correlations?**

We investigated the stability of the RDM correlations across multiple sample sizes. Correlations between RDMs were computed at cumulative intervals, where the sample size of participants increased by 10 until reaching 300 (i.e., iterations from 10-300 in steps of 10). For each step, we carried out 20 iterations. In each iteration, the required number of participants were randomly sampled from the total number of participants and computed all the same RDMs and correlation analyses as in the main manuscript. For example, for 50 participants, we conducted 20 iterations; for each iteration, we randomly sampled 50 participants from the total number of participants; we then computed three 50-by-50 RDMs, one for each variable: RDM Self-rated-social-traits, RDM Personality-traits, and RDM Face-judgements; we vectorised the RDMs and computed correlations between RDM Face-judgements and RDM Self-rated-social-traits, and between RDM Face-judgements and RDM Personality-traits; correlations were then averaged across the 20 iterations for this sample size. We repeated this procedure for each sample size from 10 to 300.

Correlations between RDM Self-rated-social-traits with RDM Face-judgements and correlations between RDM Personality-traits with RDM Face-judgements became stable with around 120 participants (**Figure S4-1**). From this, it is apparent that a sample size between 120 and 160 participants may be sufficient. It is also clear that the results are not just dependent on our large sample size, nor are they dependent on only a few participants.

**Figure S4-1**

*RDM correlations across different sample sizes.*


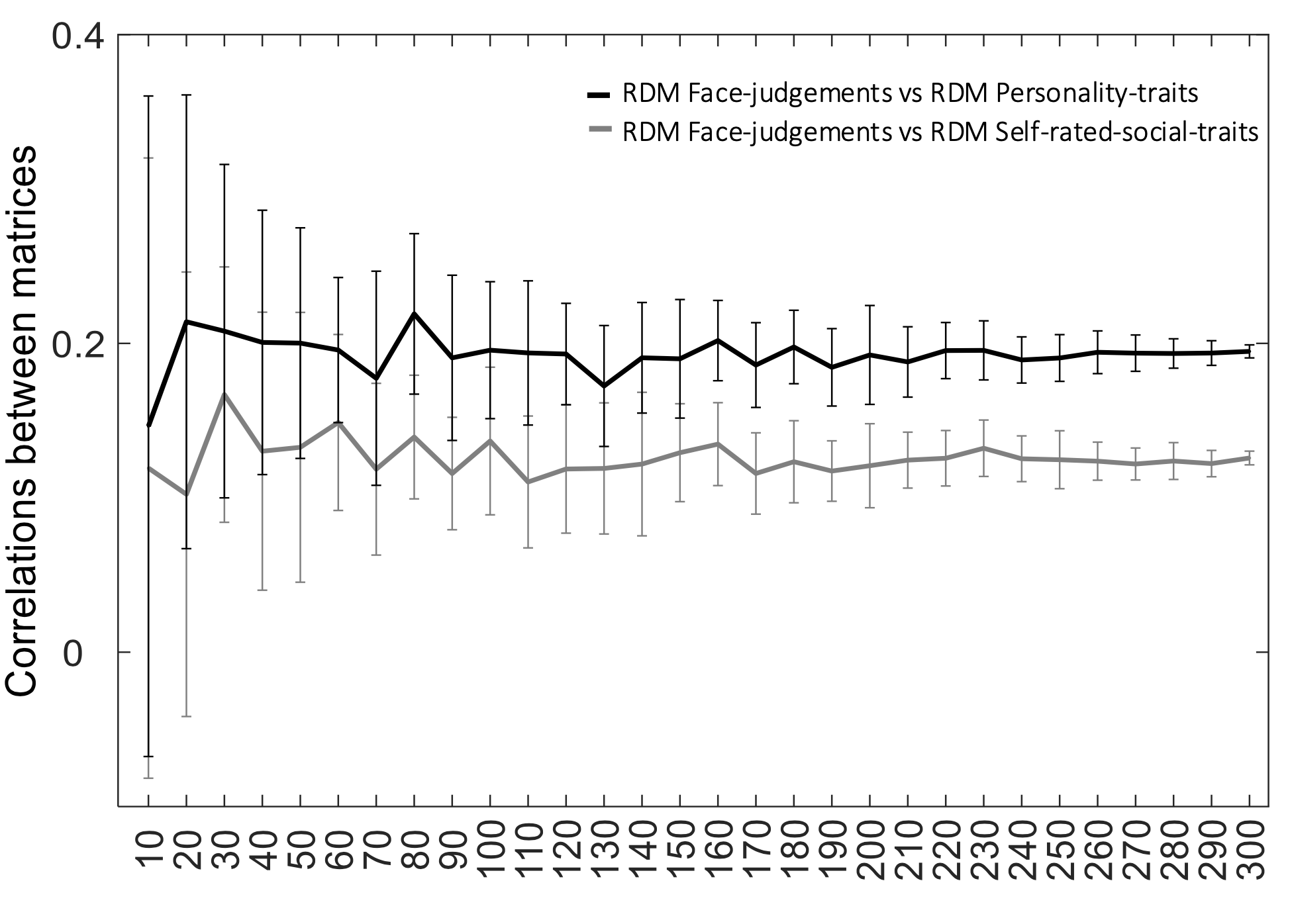


*Note.* The plot shows the correlation results with different numbers of participants. For each iteration, a random sample of participants was selected (N specified in the x-axis), RDMs were computed, and the correlation analyses were carried out. This process was carried out cumulatively, starting with a sample size of 10 participants until 300 participants. The lines show mean correlations for each sample size and the error bars show standard deviations across samples for the same N.

**Supplementary Information 5**

**Does person similarity predict face-judgements similarity for specific traits?**

We investigated whether the association of person similarity with face-judgements similarity is stronger for some face-judgements than others, (e.g., the correlation between person similarity and face *trustworthiness* judgements, specifically). It could be the case that consistencies in people’s dispositions are reflected in certain traits more strongly than in others. In other words, can we use person similarity to predict face-judgements similarity for all of the social traits? One problem with doing these correlations separately is that there are fewer trials per condition, so the sizes of these correlations cannot be directly compared to the ones using all traits simultaneously. In the analyses below, the person RDMs (RDM Self-rated-social-traits and RDM Personality-traits) were the same as in the main manuscript. However, we computed multiple RDM Face-Judgements, one for each of the face-judgements in the Face-judgements Task (Trustworthiness, Dominance, Attractiveness, Sociability, Aggressiveness and Intelligence).

Correlations between RDM Self-rated-social-traits and RDMs for each of the face-judgements: Results revealed that there were small but significant positive correlations between RDM Self-rated-social-traits and each of the individual face-trait RDMs, namely for RDM Face-trustworthiness (*r_s_* = .038, *p* < .001, random permutations), RDM Face-dominance (*r_s_* = .045, *p* < .001), RDM Face-attractiveness (*r_s_* = .067, *p* < .001), RDM Face-sociability (*r_s_* = .061, *p* < .001), RDM Face-aggressiveness (*r_s_* = .011 , *p* = .005), and RDM Face-intelligence (*r_s_* = .057, *p* < .001).

Correlations between RDM Personality-traits and RDMs for each of the face-judgements: For the correlations between RDM Personality-traits and the RDMs of each face-traits, results revealed that there were also small but positive significant correlations with the RDMs for all face traits, namely for RDM Face-trustworthiness (*r_s_* = .067, *p*  < .001), RDM Face-dominance (*r_s_* = .072, *p*  < .001), RDM Face-attractiveness (*r_s_* = .11, *p* < .001), RDM Face-sociability (*r_s_* = .090, *p*  < .001), RDM Face-aggressiveness (*r_s_* = .036, *p* < .001), and RDM Face-intelligence (*r_s_* = .058, *p* < .001).

These results showed that self-rated-social-traits similarity and personality-traits similarity could also predict similarity of trait ratings for each of the traits individually, and were not driven by any specific face-judgements. The highest correlations were between personality-traits similarity and face-judgements similarity for attractiveness and sociability.

**Supplementary Information 6**

**How do individual differences in person traits predict mean ratings of faces?**

We conducted further analyses investigating the extent to which individual differences in specific self-rated-social-traits and personality-traits contribute to the *average* face judgements. Specifically, we wanted to understand whether scoring higher or lower on each of the personality-traits and self-rated-social-traits is correlated with mean ratings of the face judgements for each trait. For example, if someone rates themselves as more trustworthy, are they more likely to rate others as more trustworthy? Matarozzi et al. (2015) had found that individuals who score higher on Agreeableness tend to rate faces as more trustworthy. Here, we extended these analyses to all traits measured. Specifically, for each participant, we computed: (i) individual scores for each of the 13 self-rated-social-traits (based on the Self-rated-social-traits Questionnaire), (ii) individual scores for each of the Big Five personality-traits (based on the Personality-traits Questionnaire), and (iii) mean ratings for each social trait on the Face-judgements Task (by computing the mean of all face ratings for each trait for the same participant). We then correlated measures in (i) and (iii), and (ii) and (iii) (**Figure S6-1**). We corrected for multiple comparisons using False Discovery Rate (FDR) correction.

Regarding correlations between individual differences in self-rated-social-traits and mean face-judgements (Figure S6-1a), there was only a significant positive correlation between self-rated-aggression and face-aggression. With regards to correlations between individual differences in personality-traits (Big 5 scores) and mean face-judgements (Figure S6-1b), there were no significant correlations after correction for multiple comparisons. These results demonstrate that our main findings of face-judgements similarity being predicted by person similarity cannot be fully explained by associations of any specific person-traits with specific face-judgements, and instead seem to reflect *patterns* of responses across traits.

**Figure S6-1**

*Correlations between individual differences in self-perceived traits (social traits and personality) and mean face judgements*


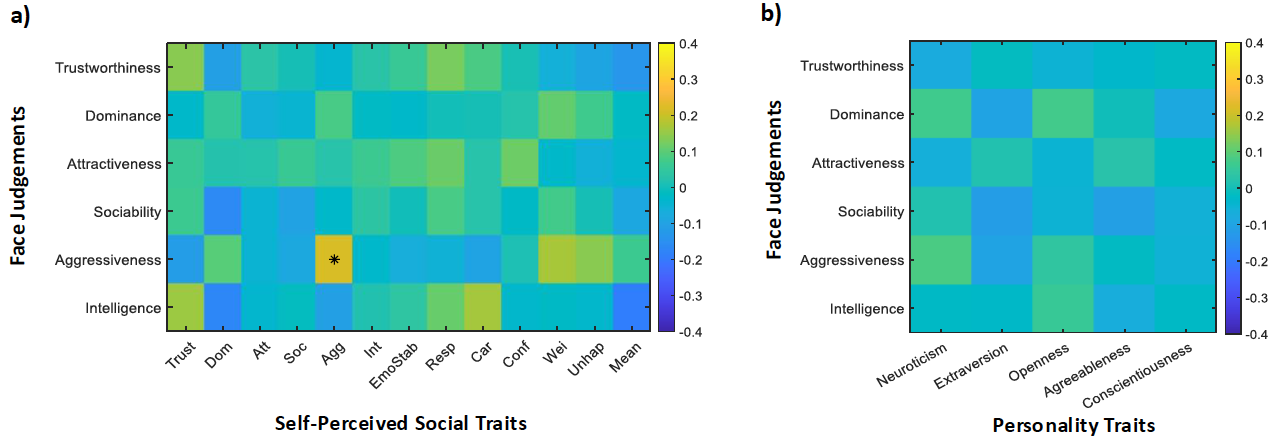


*Note.* **(a)** Correlations between individual differences in 13 self-rated-social-traits ratings and mean face-judgements for each of the 6 social traits. Trust = Trustworthiness; Dom = Dominance; Att = Attractiveness; Soc = Sociability; Agg = Aggressiveness; Int = Intelligence; EmoStab = Emotional Stability; Resp = Responsibility; Car = Caring; Conf = Confidence; Wei = Weird; Unhap = Unhappy. **(b)** Correlations between individual differences in each of the Big-5 personality-traits and mean face-judgements for each of the 6 social traits. The asterisk (*) indicates significant correlations after FDR correction for multiple comparisons.

We did not replicate the significant positive correlation between agreeableness and trustworthiness, but our sample was smaller than the one in Mattarozzi et al. (2015) and we controlled for multiple comparisons of all traits. In any case, our results suggest that individual differences in person traits are not associated only with specific changes in means of face-judgements, but instead with changes in *patterns* of judgements across many traits.

**Supplementary Information 7**

**How much do participants agree on the face-judgements?**

We next computed inter-rater agreement of face-judgements to investigate agreement of face ratings across participants. We computed inter-rater agreement in two ways. First, we computed inter-rater agreement by using a ‘pairwise approach’, in which we computed the correlation of the ratings of each participant with the ratings of each of the other participants. Second, we computed inter-rater agreement by using a ‘one *versus* others approach’ by computing the correlation of the ratings of each participant with the average of all the other participants. We computed these measures for all trials of the Face-judgements Task and also per trait (i.e., separately for Trustworthiness, Dominance, Attractiveness, Sociability, Aggressiveness and Intelligence). Results in **Table S7-1** show low to moderate inter-rater agreement for all traits and per trait, and these results are comparable to ones from previous studies (e.g. Oosterhof & Todorov, 2008), and again demonstrate that there are substantial individual differences in face-judgements. Attractiveness was the trait with the highest inter-rater agreement.

**Table S7-1**

*Inter-rater agreement for the Face-judgement Tasks (all traits and per face trait)*

|  | Mean inter-rater agreement (SD) | |
| --- | --- | --- |
| Face Judgement Task | Pairwise | One vs others |
| All traits (all trials)  Trustworthiness  Dominance  Attractiveness  Sociability  Aggressiveness  Intelligence | .23(.08)  .13(.08)  .16(.09)  .28(.10)  .22(.09)  .14(.08)  .19(.09) | .48(.16)  .36(.22)  .39(.22)  .50(.20)  .44(.20)  .39(.23)  .42(.21) |

*Note.* In the ‘pairwise approach’, we computed the correlation of the ratings of each participant with the ratings of each of the other participants. In the ‘one *versus* others approach’, we computed the correlation of the ratings of each participant with the average of all the other participants.

**References**

Mattarozzi, K., Todorov, A., Marzocchi, M., Vicari, A., & Russo, P. M. (2015). Effects of gender and personality on first impression. *PloS One*, *10*(9), e0135529.

Oosterhof, N. N., & Todorov, A. (2008). The functional basis of face evaluation. *Proceedings of the National Academy of Sciences*, *105*(32), 11087-11092.
